# Balancing Sustainability and Specimen Protection

**DOI:** 10.12688/f1000research.171192.2

**Published:** 2026-01-02

**Authors:** MV Olson

**Affiliations:** 1 The Johns Hopkins BioBank, Genetic Resources Core Facility, Department of Genetic Medicine, Johns Hopkins University School of Medicine, Baltimore, Maryland, 21287, USA

**Keywords:** Biobanking, Service centers, Cryogenic storage, Vapor-phase liquid nitrogen, Ultra-low temperature freezers, Sustainability, Specimen protection, Institutional governance

## Abstract

**Background:**

Biobanks are critical infrastructures for biomedical research but are energy- and cost-intensive due to reliance on ultra-low temperature (ULT) storage and redundant systems. The challenge is reducing environmental impact without compromising specimen quality or continuity. Service centers are well positioned to address this challenge, operating at scale and providing governance beyond the capacity of individual laboratories.

**Methods:**

The Johns Hopkins Biobank, a CAP-accredited service-center repository, partnered with the School of Medicine Energy and Sustainability Committee to conduct a freezer audit across 34 departments and two campuses. Inventories were assessed for age, utilization, and efficiency, and policies were implemented to encourage migration of biospecimens into centralized storage. Strategies prioritized vapor-phase liquid nitrogen (LN _2_) for viable collections and incorporated MVE Variō systems as energy-efficient alternatives for ULT needs. Governance required investigators to evaluate centralized options before acquiring new freezers, reinforced through outreach at faculty meetings and symposia.

**Results:**

The audit identified nearly 1,300 ULT freezers, with over 70% beyond their median life expectancy of 8.5 years. Consolidation of specimens into a Biobank-managed freezer farm reduced institutional energy demand and improved monitoring. LN _2_ provided stability for viable specimens, while Variō units offered adjustable storage (–20 °C to –150 °C) with minimal electricity use and no facility cooling load. Governance helped to curb uncontrolled expansion of departmental freezers, while the Biobank functioned as an emergency response resource with at-temperature backup capacity. Adoption of centralized storage has been gradual but continues to expand.

**Conclusions:**

This case study demonstrates how an academic service center can integrate sustainability, quality, and contingency planning. The Johns Hopkins Biobank illustrates that shared resources, supported by institutional governance, provide a practical framework to reduce environmental impact while ensuring uncompromising specimen protection.

## Introduction

Biobanks are critical infrastructures that support modern biomedical research by preserving and distributing high-quality biospecimens for discovery science, clinical translation, and precision medicine. The value of biobanking lies in its ability to safeguard irreplaceable specimens under highly controlled conditions, often over decades, ensuring they remain suitable for future research applications. ^1^ To achieve this, biobanks rely heavily on ultra-low temperature (ULT) freezers and liquid nitrogen (LN _2_) systems, which are among the most energy-intensive assets in academic research environments. ^2^

This creates a dual challenge. On one hand, institutions are under increasing pressure to reduce the environmental footprint of research infrastructure in alignment with broader sustainability commitments. ^3^ On the other, biobanks must maintain uncompromising standards of quality and continuity to protect biospecimens, meet regulatory requirements (e.g., CAP Biorepository Accreditation Program), and preserve trust with research participants. ^4^ Efforts to reduce energy use or rationalize equipment therefore cannot come at the expense of specimen protection or emergency readiness.

Although best practice frameworks from ISBER and CAP provide high-level guidance on sustainability and continuity planning, there is limited literature describing institutional models that successfully integrate both elements. ^4– 6^ Most published reports focus on either technical advances in freezer efficiency or broad policy calls for greener laboratories, with few examples grounded in operational realities of large academic biobanks. Unlike simple replacement of outdated equipment, this model couples infrastructure upgrades with institutional governance, yielding sustainability, quality, and contingency gains not achievable through technology refresh alone.

**Figure 1.**
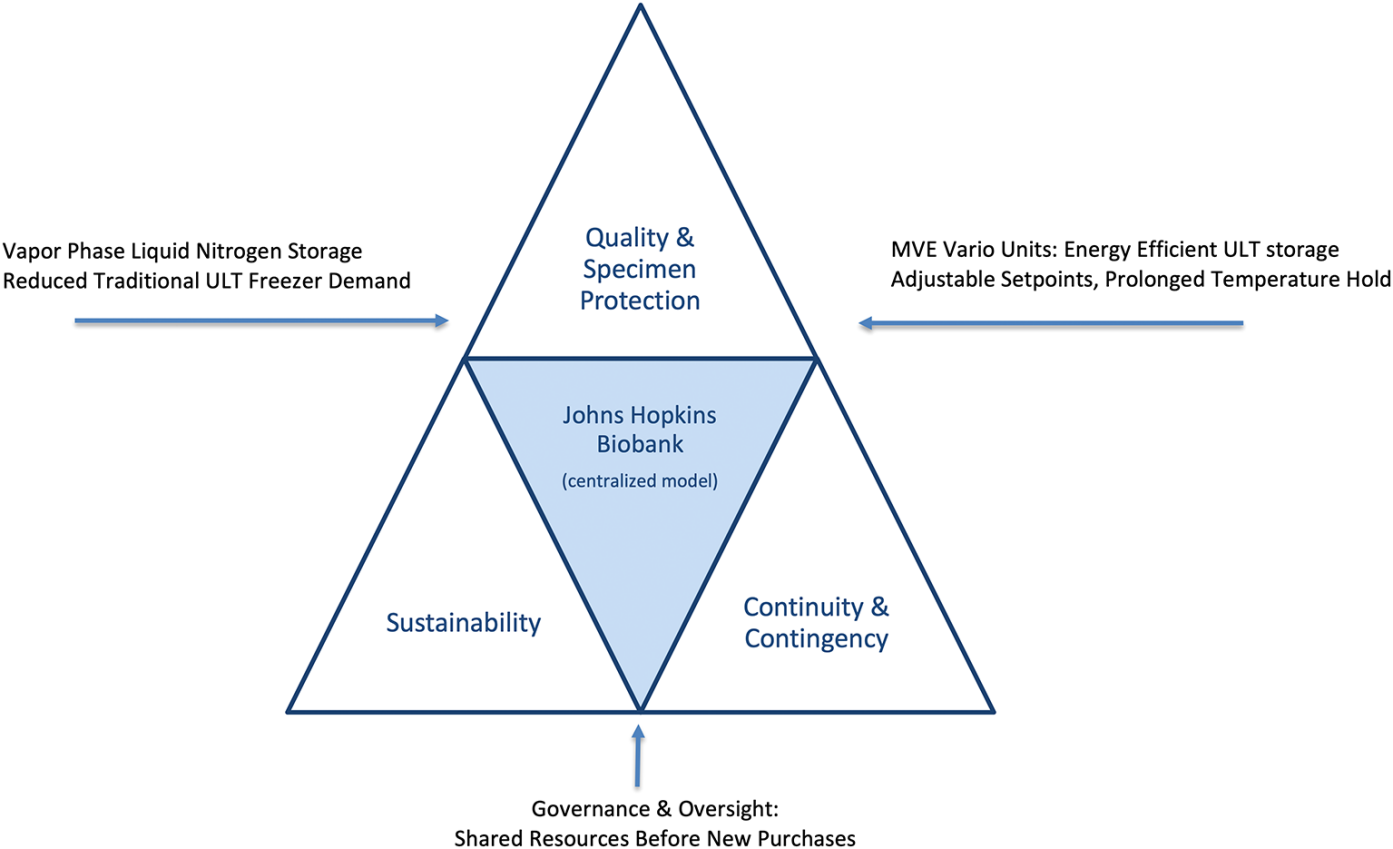
Balancing sustainability, quality, and continuity in biobanking operations. Conceptual framework illustrating how the Johns Hopkins Biobank integrates sustainability, specimen protection, and contingency preparedness. Vapor-phase liquid nitrogen (LN _2_) storage provides colder, more stable conditions for high-value specimens while reducing reliance on electricity-intensive ultra-low temperature (ULT) freezers. The MVE Vario freezer system offers an energy-efficient alternative when LN _2_ storage is not feasible, allowing flexible temperature set points with lower energy consumption and heat output. Together with centralized governance, continuous monitoring, and emergency preparedness, these strategies create a balanced, resilient infrastructure where sustainability and specimen protection reinforce rather than compete with one another.

The present case study describes the Johns Hopkins Biobank’s approach to achieving sustainability gains while strengthening specimen protection and contingency planning. Specifically, we highlight interventions in centralized freezer management, storage modality decisions, governance policies, and community engagement. By embedding sustainability within an institutional governance framework, the Biobank demonstrates that environmental responsibility and high-quality biobanking are not competing priorities, but mutually reinforcing goals ( Figure 1 and see Table 1 for the intervention framework).

**Table 1.** Key interventions implemented by the Johns Hopkins Biobank to reduce environmental impact while maintaining quality and contingency planning.

Intervention	Sustainability impact	Quality/Specimen protection	Continuity/Contingency benefit
Freezer consolidation into centralized farms	Reduced overall energy consumption and facility cooling demand	Improved oversight and consistent maintenance	Integrated monitoring and backup power ensured protection
Prioritization of vapor-phase LN _2_ storage	Lower energy footprint compared to ULT freezers	Colder storage enhances stability and longevity of specimens	Dual redundancy via LN supply contracts
Adoption of MVE Vario freezers	Energy-efficient operation with reduced heat output	Adjustable set points optimize storage conditions by sample type	Seamless integration with monitoring systems and reduced cooling loads
Retirement of inefficient legacy ULT freezers	Significant reduction in carbon footprint	Eliminated risk of failure from aging units	Simplified contingency planning
Governance policy requiring shared-resource evaluation	Prevents proliferation of unmanaged departmental freezers	Ensures proper tracking, CAP oversight, and quality assurance	Institutional alignment enhances emergency readiness
Centralized digital monitoring and 24/7 emergency response	Efficient centralized infrastructure reduces redundancies	Maintains stringent quality standards under all conditions	Immediate intervention capability during power loss or access restrictions

Summary of institutional strategies and their outcomes. Interventions included freezer consolidation, prioritization of vapor-phase LN _2_ storage, adoption of MVE Vario freezers, governance policies requiring shared-resource evaluation before new equipment purchases, and investments in centralized monitoring and emergency response. Each intervention simultaneously advanced sustainability goals, specimen protection, and institutional resilience.

## Methods/Approach

The Johns Hopkins Biobank is a College of American Pathologists (CAP)–accredited repository that operates as part of a larger service-center to support research across the Johns Hopkins University School of Medicine and its affiliated institutions. Unlike departmental freezers, which often function in isolation, the Biobank serves as a shared institutional resource: delivering high-quality storage at scale, reducing costs, and providing 24/7 monitoring and emergency response. As a service center, the BioBank is structured to support quality, rigor, and reproducibility while minimizing duplication and institutional burden.

In 2021, working with a committee on research efficiencies, the Biobank in conjunction with purchasing and institutional leadership, led an audit of freezer assets across the School of Medicine. The school includes 34 departments across two campuses (East Baltimore and Bayview) and more than 4,500 faculty. The audit identified nearly 1,300 ultra-low temperature (ULT) freezers in use, of which 938 (over 70%) were past the median life expectancy of 8.5 years. ^2^ Many operated at reduced efficiency, highlighting both the scale of energy burden and the risks associated with outdated infrastructure.

Based on these findings, new policies were established to incentivize high-efficiency replacements and to encourage migration of cohorts into centralized facilities such as the Johns Hopkins Biobank. Specimen deposits were managed as service requests, with annual storage charges applied to ensure cost neutrality and long-term sustainability of the service center. This centralized governance and shared-resource approach is depicted in the institutional model ( Figure 1). Purpose-built facilities with optimized cooling, ventilation and centralized monitoring created efficiencies that individual laboratories could not achieve.

Inventory records for all centralized collections are incorporated into the Biobank’s LIMS, supporting chain-of-custody, audit trails, and recovery planning. Associated clinical or research data remain with the investigator, consistent with consent governance and data stewardship policies. This separation ensures that enhancing physical storage does not alter data oversight or regulatory pathways.

### Governance and data handling

Governance changes were designed to improve infrastructure while preserving investigator autonomy. Sample ownership and scientific decision-making remained fully with originating research teams, regardless of storage location. The Biobank assumed responsibility for storage quality, monitoring, chain-of-custody, and 24/7 emergency response, while associated clinical and research data remained with investigators or existing institutional systems. Only minimal metadata needed for traceability (e.g., specimen type, owner, hazard status, location, global identifier) were integrated into the Biobank LIMS.

Importantly, the freezer audit revealed that research laboratories, not clinical units, accounted for nearly 80% of ULT assets, and the majority of aging, inefficient units. Historically, researchers have equated access with physical proximity, leading to highly decentralized storage. The new governance model encourages a gradual culture shift where centralized storage is prioritized for **(1)** low-access, long-term collections, **(2)** hazardous or chain-of-custody-regulated materials, and **(3)** irreplaceable or viability-dependent assets. Conversely, **daily-use research stocks and short-turnaround clinical materials** remain appropriately near the research or clinical workflow.

In efforts to reduce energy requirements within the Biobank, specimen placement strategies prioritized vapor-phase liquid nitrogen (LN _2_) storage, particularly for viable and irreplaceable collections such as cell lines and frozen tissues. For collections unsuitable for traditional vapor phase LN _2_ storage (–150 °C to –196 °C), the Biobank adopted MVE Variō freezers. These LN _2_-based units maintain user-defined set points from –20 °C to –150 °C through low energy warming mechanisms that function similar to a radiator. Further, these units operate in a dry environment with no frost or HVAC load, consume less than 1% of the electricity of mechanical ULT freezers, and reduce operating costs by ~70%. Not knowing the future needs of the Johns Hopkins community with regard to specimen storage temperatures, the Variō units were chosen as they can also be retrofitted into standard LN _2_ freezers, making them flexible assets for long-term planning. Together, LN _2_ vapor-phase and Variō systems positioned the Biobank to transition toward nitrogen-based storage solutions that conserved energy while maintaining redundancy. These complementary storage choices—LN _2_ vapor phase and Variō units—are highlighted as the primary sustainability levers in our framework ( Figure 1).

Because this initiative was intentionally aligned with natural equipment life cycles, investigators are required to evaluate community-based biobanking within the Johns Hopkins Biobank before purchasing replacements when older laboratory freezers failed. This ‘shared resources before new purchases’ policy anchors the governance element of our model ( Figure 1). Outreach campaigns, faculty meetings, and symposia reinforced awareness of centralized options and the advantages of shared stewardship. When centralized LN _2_ or Variō storage was not feasible, Biobank staff engaged in direct consultation with investigators to ensure that any new freezer purchases aligned with institutional efficiency and sustainability goals.

To safeguard continuity, the Biobank implemented centralized digital monitoring across all freezer units, with automated alerts and integration into institutional emergency protocols. Backup power, redundant LN _2_ supply, and a trained 24/7 response team provided safeguards during power outages, weather disruptions, or supply interruptions. With at-temperature storage always available, the Biobank also functioned as an emergency response unit for the Johns Hopkins community, able to accept and stabilize specimens during departmental freezer failures.

## Results & discussion

This case study demonstrates that academic biobanks can advance sustainability efforts, operational resilience, and uncompromising specimen protection within a service-center framework ( Table 1). Biobanking is often perceived as energy- and cost-intensive, yet deliberate infrastructure planning and governance reduced institutional burden while improving quality and oversight. Of the three primary objectives, sustainability benefits manifested earliest, while gains in sample protection and continuity expanded progressively as centralized infrastructure and monitoring matured.

A Johns Hopkins School of Medicine audit confirmed widespread reliance on aging, inefficient ULT freezers. Energy analyses performed during the audit demonstrated the substantial environmental burden of aging ULT freezers. Units older than 10 years showed 30–75% higher energy consumption, and those exceeding 20 years used over 100% more electricity than newer systems. Within the School of Medicine, more than 330 freezers fall within these age categories, representing significant avoidable demand on HVAC and electrical infrastructure. By contrast, LN _2_ vapor-based and Variō systems eliminate mechanical compressor load and associated cooling requirements, providing an immediately impactful sustainability mechanism as outdated freezers are retired.

Within the School of Medicine, these aging freezers consume an estimated additional $800K annually in avoidable electricity and HVAC load, based on industry-validated energy modeling ( *including manufacturer performance curves and published ULT power consumption data*). This infrastructure inefficiency is disproportionately driven by decentralized research storage, which accounts for ~80% of ULT assets across campus. These findings reinforced both the urgency and opportunity for transitioning toward centralized, nitrogen-based storage.

Adoption of centralized storage is inherently a gradual process, tied to equipment failure cycles, research timelines, voluntary participation and cultural adaptation. Most ULT freezers in academic settings are only replaced at end-of-life, and failure events are unpredictable. Accordingly, achieving full transition across eligible collections is expected to occur over multiple years rather than rapid turnover, with each unit failure representing an opportunity to migrate to sustainable, governed storage.

By working directly with departments and laboratory leads, the Biobank helped consolidate cohorts into centralized freezer farms and retire outdated units. Prioritization of LN _2_ vapor-phase and Variō storage reduced energy demand, simplified maintenance, and enhanced monitoring. Governance policies reinforced these operational changes. Investigators were required to evaluate Biobank options before replacing failed freezers, turning each replacement decision into an opportunity to expand community-based storage. When centralized Biobanking or Variō systems were not feasible, Biobank staff provided tailored consultation to guide freezer selection, ensuring that any new purchases advanced institutional sustainability goals.

Because the initiative was intentionally tied to natural equipment life cycles, adoption has been incremental rather than a single large transition. Since 2021, centralized storage within the Biobank has stabilized the equivalent capacity of approximately 42 ultra-low temperature freezers, eliminating the need for laboratories to maintain or replace those units and reducing institutional exposure to aging, high-risk infrastructure. The Biobank functions as a living system, inventory increases as new studies launch and decreases as legacy materials are dispositioned, ensuring storage capacity remains aligned with current scientific demand rather than historical accumulation. Importantly, the cost-recovery model requires investigators to review their holdings before migration, often reducing storage volume by removing obsolete or unused specimens. This cultural shift, shifting from ownership of freezers to stewardship of only what is scientifically necessary, is a central component of the sustainability and governance strategy. Rather than a one-to-one mapping of departmental assets, the evolving centralized model drives more efficient use of space, energy, and institutional oversight over time.

The Johns Hopkins model aligns with ISBER Best Practices and CAP Biorepository Accreditation Standards, which emphasize monitoring, redundancy, and quality management. Consolidating freezer assets, prioritizing LN _2_ vapor-phase storage, adopting efficient ULT technologies such as the MVE Variō, and embedding oversight into equipment purchasing translated these standards into institutional practice.

As summarized in Figure 1, the dual storage strategy was particularly impactful. LN _2_ provided unmatched protection for viable and irreplaceable specimens, while the MVE Variō offered a cost-efficient alternative for ULT collections, reducing both electricity demand and facility cooling requirements. This complementary approach maximized sample stability while minimizing financial and operational risks.

Equally important were governance and engagement. By requiring investigators to evaluate centralized options before acquiring or replacing freezers, Johns Hopkins turned each equipment failure into an opportunity to expand community-based biobanking. Where community-based storage was not possible, direct consultation ensured that institutional sustainability principles guided freezer purchases across campus. Faculty engagement through seminars and outreach further embedded shared stewardship into research culture, shifting responsibility from individual laboratories to the institution.

Although governance was strengthened through institutional oversight of storage assets, specimen ownership and decision-making authority always remain with the principal investigator. Investigators retain full control over access, use, and distribution of their collections, regardless of whether specimens are held in departmental units or in the centralized Biobank. Centralization does, however, standardize stewardship expectations, including inventory documentation, access controls, and continuity planning, ensuring that biospecimens remain protected and discoverable even as personnel or research priorities change. This model strengthens stewardship of physical materials while preserving existing data-access controls and consent protections, ensuring that participant trust and regulatory compliance remain intact. Transition risks included temporary access delays during migration, variation in laboratory readiness for inventory cleanup, and the need for stakeholder adaptation to new workflows. These were mitigated through phased adoption, **parallel storage validation,** and contingency support from the Biobank’s at-temperature backup network. No specimen losses or research disruptions occurred during the transition period.

Although this represents a single-institution experience, the principles such as auditing freezer assets, centralizing storage, prioritizing LN _2_ systems, adopting efficient ULT alternatives, embedding consultation into purchasing, and investing in monitoring and contingency—are broadly adaptable. The specific balance of centralized versus decentralized storage will differ by institution, but the framework presented here can be adapted according to local space constraints, resource availability, and regulatory environments. Academic centers facing similar pressures to improve efficiency and accountability can adopt variations of this model to strengthen both financial stability and biospecimen quality.

## Conclusion

The Johns Hopkins Biobank illustrates how sustainable practices and specimen protection can be advanced simultaneously through a service-center model. Consolidation of freezer assets, prioritization of LN _2_ vapor-phase storage, adoption of MVE Variō systems, and governance embedded into equipment replacement reduced costs, strengthened oversight, and enhanced compliance. Centralized monitoring, redundant infrastructure, and 24/7 emergency response ensured continuity of operations, while faculty outreach fostered a culture of shared stewardship. Importantly, requiring investigators to consider community-based biobanking before replacing failed freezers transformed equipment failures into opportunities for institutional strengthening.

This case provides a practical framework for other academic biobanks seeking to balance financial stability with uncompromising quality and continuity, demonstrating that efficiency and rigor can be achieved together. Ultimately, this model demonstrates that sustainability initiatives can also serve as quality initiatives, improving research rigor and resiliency while reducing institutional risk.

## Ethics statement

This work did not involve direct recruitment of human participants or the generation of new human subject data. All biospecimens referenced are managed within the Johns Hopkins Biobank under existing institutional review board approvals. The case study focuses solely on institutional infrastructure and operational practices, with no use of identifiable participant information.

## Data Availability

The analyses presented in this case study are based on internal freezer inventory records, purchasing data, and institutional operational reports from the Johns Hopkins School of Medicine. These records are considered administrative, commercially sensitive, and contain protected infrastructure information; therefore, they cannot be openly shared. The Johns Hopkins institutional compliance offices advised that operational and infrastructure datasets should remain restricted due to security and confidentiality considerations. Quantitative estimates provided in this report are based on aggregate operational assessments intended to convey institutional impact without exposing sensitive infrastructure details. Researchers who require access to these data for validation or collaborative purposes may apply through the Johns Hopkins Biobank administration. Requests will be reviewed on a case-by-case basis to ensure alignment with institutional policies on data security, confidentiality, and appropriate use. Interested parties may contact the Johns Hopkins Biobank ( biobank@jhmi.edu) for further information regarding application procedures and access conditions.
