# Supplementary material for: Balancing Sustainability and Specimen Protection
Source: F1000Res. 2026 Feb 18;14:1214. Originally published 2025 Nov 5. [Version 3] doi: 10.12688/f1000research.171192.3 (PMC12902669; doi:10.12688/f1000research.171192.3)
Supplement: Peer review history

# Reviewer report: Reviewer response for version 2

**DOI:** 10.5256/f1000research.194645.r452828

**Published:** 2026-02-12

**Authors:** Thomas F. Whale

**Affiliations:** 1 University of Leeds, Leeds, UK

**Review of “Balancing Sustainability and Specimen Protection” version 2**

 This case study describes how the Johns Hopkins Biobank approached consolidation of ultra-low temperature (ULT) storage across a large academic medical institution, aiming to reduce environmental impact while maintaining specimen quality and continuity. The intent is commendable, and the broad strategy—combining centralized governance with increased use of vapor-phase liquid nitrogen (LN₂) and energy-efficient LN₂-based systems—will be of practical interest to biobanking practitioners considering similar transitions.

 I also appreciated that the article acknowledges and seeks to manage potential pitfalls of consolidation (e.g., continuity planning, stakeholder adaptation), rather than presenting centralization as an unqualified good.

 Overall, I see this as a valuable contribution, but I think its utility would be substantially improved by a clearer definition of what is meant by “sustainability” and by quantitative framing of the claimed benefits.

 I note that I am a physical scientist with interests in cryobiology and climate science, and my comments likely reflect that perspective, which may differ somewhat from that of much of the paper’s intended audience.

**Specific comments / recommendations**

*1) Clarify what “sustainability” means, and quantify it in climate-relevant terms*

 The manuscript frames “sustainability” primarily through reduced electricity demand and HVAC load, and it emphasizes that LN₂/Variō systems use far less onsite electricity than mechanical ULT freezers. However, electricity consumption is not equivalent to carbon emissions unless the grid emissions factor is specified—and for LN₂-based strategies, the upstream energy/emissions associated with LN₂ production and supply may be material.

 If the primary motivation is greenhouse gas (GHG) emissions reduction (as the narrative implies, but does not state explicitly), I recommend adding a simple, transparent carbon accounting framework so readers can compare options on an emissions basis. Useful formats might include kgCO₂e per freezer-equivalent per year, per litre stored per year, or per sample-year. Even an illustrative worked example materially improve usefulness for institutions planning similar consolidation efforts, because it would enable comparisons aligned to climate goals rather than relying on electricity use as a proxy.

*2) Address LN₂ life-cycle emissions and allocation issues explicitly*

 Relatedly, the climate-relevant question is: what is the full system energy/emissions balance once LN₂ production, transport, and losses/boil-off are included? LN₂ may reduce onsite electricity use, but it can shift emissions “upstream” to air separation plants and distribution. A brief discussion of how these factors are (or could be) accounted for would strengthen the sustainability claim.

 There is also an allocation challenge: LN₂ is often co-produced with other industrial gases (principally oxygen), and attributing emissions between product streams is non-trivial. The manuscript would be strengthened by explicitly acknowledging this and indicating an approach even if site-specific procurement data cannot be shared.

*3) Improve evaluability and transferability with additional quantitative detail*

 The manuscript provides high-level figures (e.g., ~1,300 ULT freezers audited; >70% beyond median life expectancy; centralized capacity stabilised in “freezer-equivalent” terms), but much supporting quantitative evidence cannot be disclosed for confidentiality reasons. I appreciate that there may be institutional constraints here however, the limited quantitative detail does limit reproducibility and hence the usefulness of the case study.

 If raw data cannot be shared, I encourage adding anonymized aggregate tables plus a worked calculation that others can replicate using their own numbers. For example: assumed kWh/freezer-year by age band, assumed grid kgCO₂e/kWh, assumed LN₂ kgCO₂e/litre, and resulting kgCO₂e per freezer-equivalent-year for each storage method. Even a single representative numeric comparison between storage methods would help anchor the claims.

Is the case presented with sufficient detail to be useful for teaching or other practitioners?

Partly

Is the work clearly and accurately presented and does it cite the current literature?

Yes

If applicable, is the statistical analysis and its interpretation appropriate?

Not applicable

Are all the source data underlying the results available to ensure full reproducibility?

No

Are the conclusions drawn adequately supported by the results?

Partly

Is the background of the case’s history and progression described in sufficient detail?

Yes

Reviewer Expertise:

I am a physical chemist interested in formation of water ice. I work on applied questions in atmospheric and climate science and cryobiology.

I confirm that I have read this submission and believe that I have an appropriate level of expertise to confirm that it is of an acceptable scientific standard, however I have significant reservations, as outlined above.

# Reviewer report: Reviewer response for version 2

**DOI:** 10.5256/f1000research.194645.r452830

**Published:** 2026-02-10

**Authors:** Rajasekharan P.E.

**Affiliations:** 1 ICAR- Indian Institute of Horticultural Research, Bengaluru, Karnataka, India

The case study presents an informative account of sustainability-driven biobank consolidation; however, several limitations constrain its broader applicability and evidentiary strength. Foremost among these is the reliance on a single, exceptionally well-resourced institution. The Johns Hopkins University setting benefits from strong centralized governance, stable funding, mature infrastructure, and institutional authority that are not typical of many academic biobanks, particularly those operating in low- and middle-income countries or smaller universities. Consequently, the proposed framework, while internally coherent, risks being perceived as context-specific rather than broadly transferable, despite assertions of general applicability.

 A second major concern relates to the limited transparency of quantitative data. Although the manuscript emphasizes substantial gains in sustainability and cost efficiency, most supporting data remain inaccessible due to claims of institutional sensitivity. Key indicators such as absolute sample numbers, the proportion of collections centralized, personnel effort invested, and energy savings relative to total institutional consumption are either aggregated, estimated, or omitted. This lack of granular data significantly limits reproducibility, independent validation, and benchmarking, thereby reducing the practical utility of the study for other biobanks seeking evidence-based guidance.

 The robustness of the conclusions is further weakened by an overreliance on modeled rather than empirically measured outcomes. Reported benefits related to energy savings and financial efficiency are primarily derived from manufacturer specifications, industry-validated models, and inferred reductions in HVAC load, rather than direct pre- and post-intervention measurements. While such estimates are reasonable, the absence of longitudinal empirical data undermines the strength of claims such as large operating cost reductions and substantial avoidable annual expenditures, which are central to the manuscript’s argument.

 In addition, the discussion of risks and failure scenarios is relatively limited. Although the authors report that no specimen losses occurred, potential vulnerabilities associated with large-scale centralization—such as sample degradation during migration, access delays, dependence on uninterrupted liquid nitrogen supply, and reliance on centralized digital monitoring—are addressed only briefly. Broader systemic risks, including single-point failures, reduced redundancy, over-standardization, and possible erosion of investigator autonomy, are acknowledged but not critically examined in depth.

 The success of the model also appears heavily dependent on institutional governance mechanisms that may be difficult to replicate elsewhere. Mandatory evaluation of centralized storage prior to freezer replacement requires strong administrative authority and compliance cultures, which are often absent in decentralized academic environments. The manuscript does not sufficiently explore potential resistance from investigators, non-compliance, or enforcement challenges, all of which represent realistic barriers to implementation in less centralized institutions.

 Furthermore, sustainability is framed predominantly through energy consumption, HVAC load, and carbon footprint metrics. While these are important, the analysis gives relatively little attention to broader life-cycle considerations, including the environmental costs of liquid nitrogen production, transport emissions, nitrogen losses, and infrastructure redundancy. This narrow framing risks oversimplifying the environmental trade-offs between nitrogen-based storage systems and advanced mechanical ultra-low temperature technologies.

 The manuscript also lacks a systematic comparison with alternative or hybrid models. Centralized, LN₂-based biobanking is presented as the preferred solution without rigorous comparison to other viable strategies such as optimized decentralized storage, right-sizing of departmental freezers, AI-assisted freezer management, or next-generation energy-efficient ULT systems. As a result, the argument that the proposed approach represents the optimal pathway remains only partially substantiated.

 Finally, despite its length, the article remains largely conceptual rather than procedural. Practical implementation details—such as standardized operating procedures for sample transfer, chain-of-custody validation, redistribution of staff workloads, and management of cost-recovery tensions—are not described in sufficient detail for the study to function as an operational manual. This limits its instructional value for practitioners who may wish to replicate or adapt the model in their own institutional contexts.

 In summary, while the study offers a valuable strategic perspective on integrating sustainability with specimen protection, its impact is constrained by data opacity, limited risk analysis, governance assumptions, and restricted generalizability. The work is best viewed as a high-level institutional case narrative rather than a fully evidence-driven, universally applicable implementation framework.

Is the case presented with sufficient detail to be useful for teaching or other practitioners?

No

Is the work clearly and accurately presented and does it cite the current literature?

Yes

If applicable, is the statistical analysis and its interpretation appropriate?

No

Are all the source data underlying the results available to ensure full reproducibility?

Yes

Are the conclusions drawn adequately supported by the results?

No

Is the background of the case’s history and progression described in sufficient detail?

Yes

Reviewer Expertise:

Plant genetic resources exploration, collection, evaluation and conservation including cryoprservation

I confirm that I have read this submission and believe that I have an appropriate level of expertise to state that I do not consider it to be of an acceptable scientific standard, for reasons outlined above.

# Response

**Published:** 2026-02-11

**Authors:** Melissa Olson

**Affiliations:** Genetic Medicine, Johns Hopkins University School of Medicine, Baltimore, Maryland, USA

**Response to Reviewer**

 I thank the reviewer for their careful reading of the revised case study and for the detailed critique. I appreciate the reviewer’s recognition that the background and progression of the initiative are described in sufficient detail and that the work is clearly presented with appropriate literature citation. Several concerns appear to reflect expectations more typical of multi-site quantitative benchmarking or lifecycle assessment studies rather than an operational institutional case study. In response, I have made targeted revisions to clarify scope, methods, limitations, transferability, and risk mitigation while maintaining the intent of the manuscript as a real-world implementation narrative.

 Below I respond point-by-point.

 1) Reliance on a single, well-resourced institution / generalizability

**Reviewer concern:** The Johns Hopkins setting is unusually well resourced and therefore the framework risks being context-specific rather than broadly transferable.

**Response:** I agree that institutional context influences feasibility, governance mechanisms, and pace of adoption. This manuscript is explicitly a case study describing an implemented institutional model within a defined governance and infrastructure environment—not a universally prescriptive blueprint. To make this clear to readers, I strengthened the framing in the Abstract and in the Results & Discussion section, explicitly stating that the report “is intended to illustrate operational strategy within a defined governance and infrastructure environment rather than to function as a universally prescriptive implementation model.” *[NEW TEXT ADDED: Abstract Conclusions]*

 I also added language clarifying that while the specific governance tools available at Johns Hopkins may not be replicable everywhere, several principles (auditing, central monitoring, consolidation incentives, contingency planning, consultation embedded in replacement decisions) can be adapted using alternative local mechanisms. *[NEW TEXT ADDED: Results & Discussion transferability paragraph]*

 2) Limited transparency / lack of granular quantitative data

**Reviewer concern:** Key indicators (absolute sample numbers, proportion centralized, personnel effort, energy savings relative to total institutional consumption) are estimated, aggregated, or omitted due to institutional sensitivity, limiting reproducibility and benchmarking.

**Response:** I appreciate the reviewer’s interest in granular operational metrics. However, some detailed infrastructure-level and inventory data are subject to institutional security, confidentiality, and commercial sensitivity constraints (e.g., facility asset inventories, infrastructure load profiles, and detailed repository operational counts). To address this transparently, I strengthened the Data Availability statement to explain why open sharing is not possible and how access requests may be reviewed on a case-by-case basis for collaborative validation purposes. *[NEW TEXT ADDED/EXPANDED: Data Availability]*

 I also reinforced in the Limitations section that aggregated reporting may limit direct benchmarking, while still conveying institutional-scale operational impact. *[NEW TEXT ADDED: Limitations]*

 Importantly, the purpose of this case study is to describe a governance-enabled operational pathway linking sustainability with specimen protection and continuity planning. I have clarified this scope so readers do not interpret the manuscript as a substitute for a full institutional energy metering study or a multi-institution benchmarking dataset.

 3) Modeled vs. empirically measured outcomes (energy and cost savings)

**Reviewer concern:** Key sustainability benefits are derived from manufacturer specifications and models rather than longitudinal pre/post measurements.

**Response:** I agree that modeled outcomes differ from longitudinal metered measurements, and I have clarified this distinction explicitly in the Methods/Approach section. I added a statement noting that sustainability and energy-reduction estimates were derived from institutional equipment inventories, manufacturer performance specifications, and validated engineering models, and that direct longitudinal metering of individual freezer energy consumption was not feasible retrospectively due to shared infrastructure loads and concurrent institutional energy initiatives. *[NEW TEXT ADDED: Methods/Approach]*

 In addition, I refined language describing Variō performance to indicate these are projected operating cost reductions based on manufacturer specifications and published benchmarks. *[NEW TEXT ADDED: Governance and Data Handling section]*

 I believe these clarifications appropriately bound the evidentiary strength of the estimates while maintaining the practical relevance of the institutional planning approach described.

 4) Risk analysis / failure scenarios of large-scale centralization

**Reviewer concern:** Risks such as migration-related degradation, access delays, LN₂ dependence, single-point failures, and monitoring dependence are not examined in depth.

**Response:** I agree that risk mitigation is central to centralization initiatives. In response, I expanded discussion of transition risks and controls, including phased adoption, parallel validation, contingency support, centralized monitoring, redundant LN₂ supply, and 24/7 response capability. I also explicitly note that transition risks included temporary access delays and stakeholder adaptation, and that these were mitigated through structured implementation safeguards, with no specimen losses or research disruptions observed during the transition period. *[NEW TEXT ADDED/EXPANDED: Results & Discussion risk mitigation paragraph]*

 5) Governance assumptions / investigator resistance and enforcement challenges

**Reviewer concern:** Mandatory evaluation of centralized storage depends on governance authority that may be difficult to replicate; the manuscript does not sufficiently explore resistance or compliance barriers.

**Response:** I agree that governance culture varies widely and that identical policy mechanisms are not universally transferable. I clarified that implementation mechanisms will necessarily vary by institutional size, infrastructure maturity, and resource availability, and that adoption occurs gradually through equipment life cycles, voluntary participation, and cultural adaptation. *[NEW TEXT ADDED: Results & Discussion adoption and transferability paragraphs]*

 I also added explicit language acknowledging that investigator adoption varied and required ongoing engagement, transparency around cost structures, and demonstration of specimen stewardship benefits to sustain participation. *[NEW TEXT ADDED: Results & Discussion adoption paragraph]*

 6) Sustainability framing focused largely on electricity/HVAC rather than lifecycle impacts of LN₂

**Reviewer concern:** Sustainability is treated narrowly; environmental trade-offs of LN₂ production/transport are not examined.

**Response:** I agree lifecycle considerations are important. The institutional audit and sustainability framework described here focused on electrical demand and HVAC load because these were the actionable, measured, and decision-relevant drivers within the institutional planning context at the time of implementation. To acknowledge the broader scope, I added a statement noting that lifecycle considerations (including LN₂ production and transport) are relevant sustainability dimensions that warrant future evaluation but were outside the operational scope of the institutional audit described. *[NEW TEXT ADDED: Results & Discussion sustainability paragraph]*

 7) Limited comparison with alternative or hybrid models

**Reviewer concern:** LN₂-based centralization is presented as preferred without rigorous comparison to optimized decentralized storage or next-generation ULT systems.

**Response:** This manuscript documents the implemented institutional strategy rather than conducting a comparative trial across all possible sustainability pathways. That said, I agree multiple viable approaches exist depending on institutional context. I have clarified that the paper should be interpreted as a case narrative describing a governance-enabled service-center pathway, not as a claim of universal optimality across all settings. Added generalizability language emphasizes adaptation rather than prescriptive superiority. *[REFLECTED IN: Abstract scope statement; Results & Discussion institutional context and transferability language]*

 8) The article is conceptual rather than procedural; limited “how-to” detail

**Reviewer concern:** Insufficient procedural detail to function as a replication manual (SOPs for transfer, chain-of-custody validation, workload redistribution, cost-recovery tensions).

**Response:** I agree that a step-by-step operational manual could be valuable; however, such procedural documents are necessarily institution-specific and can be extensive (and in many cases are not publishable in full due to infrastructure security and internal policy constraints). This manuscript is intentionally framed as a case study highlighting governance mechanisms, storage modality decisions, and an intervention framework (Figure 1; Table 1) rather than as a comprehensive operational SOP compendium.

 To increase practical utility without expanding the manuscript beyond case-study scope, I strengthened descriptions of governance, chain-of-custody integration in the Biobank LIMS, and transition risk mitigation. *[NEW TEXT ADDED/EXPANDED: Methods/Approach; Governance and Data Handling; Results & Discussion]*

 9) Statistical analysis and reproducibility checklist items

**Reviewer concern:** “Statistical analysis is appropriate: No” and concerns regarding reproducibility.

**Response:** I respectfully note that this manuscript is an operational case study and does not present hypothesis-driven experimental comparisons requiring statistical testing. The analyses are descriptive and planning-oriented, based on institutional inventory audits and standard sustainability planning estimates. I have clarified the methodological basis and limitations of modeled estimates, and strengthened the Data Availability statement to transparently explain constraints and pathways for controlled access where appropriate.

 Closing

 I again thank the reviewer for their detailed feedback. I believe the targeted revisions improve clarity regarding scope, evidentiary boundaries, transferability, and risk mitigation while preserving the manuscript’s purpose: to document a real-world, governance-enabled institutional approach to integrating sustainability with specimen protection and continuity planning in an academic service-center biobank.

# Reviewer report: Reviewer response for version 2

**DOI:** 10.5256/f1000research.194645.r446685

**Published:** 2026-01-22

**Authors:** Veit Braun

**Affiliations:** 1 Institute for Social Sciences, Universitat Augsburg Philosophisch-Sozialwissenschaftliche Fakultat (Ringgold ID: 235824) Place: Augsburg, Augsburg, Bavaria, Germany

This is a much improved version of the report, thank you very much for your revisions and additions. Most of the additional information I would have liked to see in the article is now included and I think in its current form it makes for a useful reading for biobanking practitioners. I recommend that the revised version be accepted.

Is the case presented with sufficient detail to be useful for teaching or other practitioners?

No

Is the work clearly and accurately presented and does it cite the current literature?

Partly

If applicable, is the statistical analysis and its interpretation appropriate?

Not applicable

Are all the source data underlying the results available to ensure full reproducibility?

No

Are the conclusions drawn adequately supported by the results?

Partly

Is the background of the case’s history and progression described in sufficient detail?

Partly

Reviewer Expertise:

Organization studies, sociology of science and technology, environmental studies

I confirm that I have read this submission and believe that I have an appropriate level of expertise to confirm that it is of an acceptable scientific standard.

# Reviewer report: Reviewer response for version 1

**DOI:** 10.5256/f1000research.188763.r435173

**Published:** 2025-12-30

**Authors:** Veit Braun

**Affiliations:** 1 Institute for Social Sciences, Universitat Augsburg Philosophisch-Sozialwissenschaftliche Fakultat (Ringgold ID: 235824) Place: Augsburg, Augsburg, Bavaria, Germany

This article offers an interesting case study of monitoring a centralized biobank by a service center of one of the United States' leading medical research institutions (Johns Hopkins University Medical School).

 The text gives some basic results of a survey among 34 departments, which together ran about 1,300 ultra-low temperature freezers, over 900 of which had exceeded their median life expectancy and were subsequently phased out. Where possible, these freezers were replaced by more cost- and energy-efficient liquid nitrogen freezers and LN-based Vario freezers. The author claims that considerable cost savings, energy intensity reductions as well as preservation and planning improvements (contingency and continuity) were achieved in the process.

 Unfortunately no case-specific data are provided beyond the basic figures of 34 departments, 1,300 ULT freezers and 938 units beyond their median life expectancy. While the basic argument - an audit process leading to organisational improvements - is highly plausible, it is thus difficult to evaluate.

 Out of the three key areas of sustainability, sample quality and continuity/contingency, which benefitted most from the audit? What was the survey's response rate - were there institutions/facilities which it was difficult to obtain data on (and if so, why)? Where were improvements most difficult to achive because centralized and decentralized storage facilities were already close to an optimum? What is the approximate ballpark of savings and improvements, especially when put in relation to Johns Hopkins's overall biobanking facilities? What sample types/uses required Vario freezers or decentralized storage as a bespoke solution, and what percentage of the replaced units/relocated samples did the latter account for? How did the transitioning process look like in practice: concerted bulk transfer of samples into new units or unit-by-unit transfer? What does the protocol for picking up samples at one of the departments and transfering them to Johns Hopkins Biobank look like? Were potential downsides to centralized storage identified over the process?

 I believe these are questions the article should answer if it is supposed to inform the reader about the potentials of centralized audits and storage services. The author points out that most of the underlying data is sensitive and thus cannot be made public. I agree that this applies to items such as budget, location, internal decision-making, personal data and some of the risks identified during the audit.

 A number of items could be shared as data or information in the text at least in very general form. In my view, these include:

 - Overall number of samples and units in Johns Hopkins Biobank and the share of decentralized samples and units that remain after the audit. This does not identify sample types, locations or persons but allows the reader to infer organizational impact of the process described.

 - Sample types that require bespoke or decentralized solutions. No exhaustive list and no very fine-grained details need to be given - "cell lines" (as example) or "samples for daily/weekly use" (as a descriptor) would suffice for readers to assess whether a similar process would work for their own biobank(s).

 - A very rough estimate (as a share of total energy consumed over a certain span of time) of energy savings achieved by the audit

 - The overall number of staff/person hours involved in the process. This will allow readers to weigh costs of the audit against potential savings in their own facilities.

 - A very general list of risks occuring at the various stages of the process (e.g. sample quality, data migration, relocation too few or too many samples, drawbacks of centralized storage and reduced redundancies that require additional precautionary measures) should be given to prepare readers for the contingencies along the way. No specific examples that were involved in the study need to be given here but a list of eventualities would be very useful.

 I would argue that these data can be made publicly available without a case-by-case negotiation with Johns Hopkins Biobank, which adds a rather steep hurdle (delayed response, bargaining, refusal, transfer and use agreements) for those seeking to replicate the success of the audit (and Johns Hopkins Biobank, too), not least in person hours.

 Furthermore, the article should address one very central point: Owing to technological progress, one would assume improvements in at least one of the targeted key areas mentiones by the author while the other two remain at their previous performance levels or are improved as well. A critical reader could thus object that any unit replaced by an improved model would fulfil the goals outlined in Fig. 1. What additional benefits does the centralized auditing process provide over the mere replacement of old, inefficient technology?

 In my view, it is that such a centrally planned process allows for pooling resources and thus complying with individual and overall budget and other resource constraints. Costs would otherwise both exceed departments' budgets (new units are too expensive) and redundancies would increase overall (new units are under-utilized when decentrally installed; freezer space will not be fully used). New technology positively addresses all three goals but improvements need to be weighed against expenses and an optimal tradeoff should be identified over the course of the internal planning process. If this is indeed the reasoning behind the audit, the article should state it more clearly early on.

 In this case, it should also explain under what criteria, very generally speaking, the biobank deviated from the goal of cost and energy savings. As a medical institution, I would expect that Johns Hopkins prioritizes sample quality and continuity if in doubt, ie, centralized storage was deprioritized in those cases where sample quality and continuity were more likely to be achieved with decentralized and bespoke solutions (if at higher costs). Should that be the case, the author should briefly lay out the general reasoning and prioritization in balancing the goals of the biobank.

 If the author is able to address these issues and questions to a substantial degree I am convinced the report will make for a useful template for other biobanks.

Is the case presented with sufficient detail to be useful for teaching or other practitioners?

No

Is the work clearly and accurately presented and does it cite the current literature?

Partly

If applicable, is the statistical analysis and its interpretation appropriate?

Not applicable

Are all the source data underlying the results available to ensure full reproducibility?

No

Are the conclusions drawn adequately supported by the results?

Partly

Is the background of the case’s history and progression described in sufficient detail?

Partly

Reviewer Expertise:

Organization studies, sociology of science and technology, environmental studies

I confirm that I have read this submission and believe that I have an appropriate level of expertise to state that I do not consider it to be of an acceptable scientific standard, for reasons outlined above.

# Response

**Published:** 2025-12-30

**Authors:** Melissa Olson

**Affiliations:** Genetic Medicine, Johns Hopkins University School of Medicine, Baltimore, Maryland, USA

**Response to Reviewer (Dr. Veit Braun)**

 I am grateful for the reviewer’s careful reading of the manuscript and for the constructive, detailed suggestions. Below, I address each of the major points and indicate the corresponding revisions made to the manuscript.

**Direct Responses:**

**1. “No case-specific data… difficult to evaluate. Which of the three areas benefited most?”**

**Reviewer comment (paraphrased)**

 The manuscript does not provide sufficient case-specific data beyond 34 departments, ~1,300 ULTs, and 938 units beyond median life expectancy. It is difficult to evaluate the impact. Out of the three key areas—sustainability, sample quality, and continuity/contingency—which benefitted most?

**Response**

 I appreciate this concern and have revised the manuscript to provide more specific, yet still appropriately aggregated, institutional data.

- In the Results & discussion section, I now explicitly state which dimension showed the earliest, most measurable gains: “Of the three primary objectives, sustainability benefits manifested earliest, while gains in sample protection and continuity expanded progressively as centralized infrastructure and monitoring matured.”

- I have added quantitative estimates of the additional energy burden associated with aging freezers and the scale of centralized capacity that has been stabilized within the Biobank: “Energy analyses performed during the audit demonstrated the substantial environmental burden of aging ULT freezers. Units older than 10 years showed 30–75% higher energy consumption, and those exceeding 20 years used over 100% more electricity than newer systems. Within the School of Medicine, more than 330 freezers fall within these age categories, representing significant avoidable demand on HVAC and electrical infrastructure.”- and- “Within the School of Medicine, these aging freezers consume an estimated additional $800K annually in avoidable electricity and HVAC load, based on industry-validated energy modeling (including manufacturer performance curves and published ULT power consumption data).”- and- “Since 2021, centralized storage within the Biobank has stabilized the equivalent capacity of approximately 42 ultra-low temperature freezers, eliminating the need for laboratories to maintain or replace those units and reducing institutional exposure to aging, high-risk infrastructure.”

Together, these additions clarify both the relative contribution of the sustainability dimension and the scale of change achieved, while maintaining institutional confidentiality.

**2. “Overall number of samples/units and share remaining decentralized; sample types requiring bespoke or decentralized solutions”**

**Reviewer comment (paraphrased)**

 The reviewer requests the overall number of samples and units in the Biobank and the share that remain decentralized, as well as more detail on which sample types require bespoke or decentralized storage (e.g. daily-use materials, cell lines, etc.).

**Response**

 I agree that readers need a clearer understanding of which collections are appropriate for centralization versus decentralization. For security and confidentiality reasons, I do not disclose absolute institutional sample counts, but I have strengthened the description of proportional impact and sample categories.

- In Governance and Data Handling, I now clarify where the greatest burden lies and which broad classes of material are targeted for centralization: “Importantly, the freezer audit revealed that research laboratories, not clinical units, accounted for nearly 80% of ULT assets, and the majority of aging, inefficient units. Historically, researchers have equated access with physical proximity, leading to highly decentralized storage. The new governance model encourages a gradual culture shift where centralized storage is prioritized for (1) low-access, long-term collections, (2) hazardous or chain-of-custody-regulated materials, and (3) irreplaceable or viability-dependent assets. Conversely, daily-use research stocks and short-turnaround clinical materials remain appropriately near the research or clinical workflow.”

- In the Results & discussion, I further explain that centralization is not a one-to-one mapping of all existing units but an opportunity to right-size inventories: “The Biobank functions as a living system, inventory increases as new studies launch and decreases as legacy materials are dispositioned, ensuring storage capacity remains aligned with current scientific demand rather than historical accumulation. Importantly, the cost-recovery model requires investigators to review their holdings before migration, often reducing storage volume by removing obsolete or unused specimens. This cultural shift, shifting from ownership of freezers to stewardship of only what is scientifically necessary, is a central component of the sustainability and governance strategy.”

These revisions clarify both the relative contribution of research versus clinical storage and the types of samples that are preferentially centralized, without exposing detailed inventory figures that are considered sensitive institutional data.

**3. “Approximate energy savings; added value of centralized audit vs simple technology replacement”**

**Reviewer comment (paraphrased)**

 What is the approximate ballpark of energy savings? Also, what additional benefits does centralized auditing and governance provide beyond simply replacing old ULTs with more efficient models?

**Response**

 I fully agree that readers must understand both the magnitude of the problem and why a governance-based approach is superior to simple equipment replacement.

- To address the energy/financial aspect, the Results & discussion now includes: “Within the School of Medicine, these aging freezers consume an estimated additional $800K annually in avoidable electricity and HVAC load, based on industry-validated energy modeling (including manufacturer performance curves and published ULT power consumption data).”

This provides a concrete, institution-level estimate of avoidable cost and energy burden.

- To make the added value of centralized governance explicit early in the manuscript, I added to the Introduction: “Unlike simple replacement of outdated equipment, this model couples infrastructure upgrades with institutional governance, yielding sustainability, quality, and contingency gains not achievable through technology refresh alone.”

- Throughout the Results & discussion, I emphasize that the central audit and governance framework:
  - prevents proliferation of unmanaged departmental freezers,
  - ties replacement decisions to shared-resource evaluation,
  - enforces monitoring, redundancy and continuity planning,
  - and drives inventory cleanup and right-sizing (rather than simply upgrading the same number of under-utilized units).

In this way, I have made the conceptual distinction between “better technology” and “better governance plus technology” much clearer.

**4. “Which areas benefited most, where were improvements difficult, and how was the transition operationalized?”**

**Reviewer comment (paraphrased)**

 Which of sustainability, sample quality, and continuity benefitted most? Where were improvements hardest because centralized and decentralized storage were already near an optimum? What did the transitioning process look like in practice—bulk vs unit-by-unit transfers, and what is the protocol for picking up samples?

**Response**

 I have clarified both the differential timing of benefits and the practical nature of the transition:

- As noted under Point 1, I now state explicitly: “Of the three primary objectives, sustainability benefits manifested earliest, while gains in sample protection and continuity expanded progressively as centralized infrastructure and monitoring matured.”

- Regarding where improvements are most difficult, the Governance and Data Handling section makes explicit that high-frequency, short-turnaround materials remain decentralized by design: “…centralized storage is prioritized for (1) low-access, long-term collections, (2) hazardous or chain-of-custody-regulated materials, and (3) irreplaceable or viability-dependent assets. Conversely, daily-use research stocks and short-turnaround clinical materials remain appropriately near the research or clinical workflow.”

This clarifies that for heavily used, workflow-critical materials, the system is already close to an operational optimum and centralization is not pursued.

- The gradual, failure-aligned transition process is now emphasized in Methods/Approach and Results & discussion: “Because this initiative was intentionally aligned with natural equipment life cycles, investigators are required to evaluate community-based biobanking within the Johns Hopkins Biobank before purchasing replacements when older laboratory freezers failed.”- and- “Adoption of centralized storage is inherently a gradual process, tied to equipment failure cycles, research timelines, voluntary participation and cultural adaptation. Most ULT freezers in academic settings are only replaced at end-of-life, and failure events are unpredictable. Accordingly, achieving full transition across eligible collections is expected to occur over multiple years rather than rapid turnover, with each unit failure representing an opportunity to migrate to sustainable, governed storage.”

- Transfers are predominantly done at the level of defined cohorts, coordinated with investigators and Biobank staff, rather than a one-time bulk move of the entire institution. While a highly detailed step-wise protocol would be institution-specific and beyond the scope of this case study, I now describe the high-level approach as a phased, cohort-level migration tied to failure events and investigator readiness, supported by the Biobank’s 24/7 emergency response capacity.

**5. “Sample types needing Vario or decentralized storage; percentage of replaced units/samples”**

**Reviewer comment (paraphrased)**

 What sample types or uses required Vario freezers or decentralized storage, and approximately what percentage of replaced units or relocated samples did these represent?

**Response**

 I have clarified the sample-type rationale, but I have not provided exact percentages, as those data are sensitive and not easily de-identified without risk of revealing institutional structure.

- In Governance and Data Handling, as quoted above, I now specify three categories of material prioritized for centralization and note explicitly that “daily-use research stocks and short-turnaround clinical materials” remain near the research or clinical workflow.
- In the Methods/Approach, I also emphasize the functional differentiation between LN₂ and Variō units: “In efforts to reduce energy requirements within the Biobank, specimen placement strategies prioritized vapor-phase liquid nitrogen (LN2) storage, particularly for viable and irreplaceable collections such as cell lines and frozen tissues. For collections unsuitable for traditional vapor phase LN2 storage (–150 °C to –196 °C), the Biobank adopted MVE Variō freezers… These complementary storage choices—LN2 vapor phase and Variō units—are highlighted as the primary sustainability levers in our framework (Figure 1).”

Thus, while I cannot provide precise percentages of relocated sample types, readers now have a clear rationale for which materials are best suited to LN₂, to Variō systems, and to remaining decentralized.

**6. “Staff/person-hours and costs of the audit; ability to generalize”**

**Reviewer comment (paraphrased)**

 The reviewer asks for the overall number of staff/person-hours involved to allow readers to weigh costs of the audit against potential savings.

**Response**

 I agree that staff effort is an important consideration. However, detailed time tracking was not performed at the level required for a robust quantitative estimate. The audit and implementation were performed by existing Biobank and institutional sustainability staff as part of their ongoing responsibilities, rather than as a separately budgeted project with dedicated FTE lines. Rather than provide a speculative numeric estimate, I have chosen to describe the work qualitatively as an institutional initiative executed within existing organizational structures.

 If the editor feels that even a qualitative statement about staffing would be helpful, I would be glad to add a sentence such as: “The audit and subsequent policy implementation were conducted by existing Biobank and institutional sustainability staff within their routine roles, without creation of dedicated full-time positions.”

 I hope this approach balances transparency about resource requirements with the limitations of available data.

**7. “Risks and downsides to centralized storage; list of contingencies”**

**Reviewer comment (paraphrased)**

 A general list of risks at various stages (sample quality, data migration, over- or under-relocation, drawbacks of centralized storage) would be useful, along with any potential downsides identified.

**Response**

 I appreciate this suggestion and have added explicit discussion of risks and mitigation strategies in the Results & discussion: “Transition risks included temporary access delays during migration, variation in laboratory readiness for inventory cleanup, and the need for stakeholder adaptation to new workflows. These were mitigated through phased adoption, parallel storage validation, and contingency support from the Biobank’s at-temperature backup network. No specimen losses or research disruptions occurred during the transition period.”

 In addition, the Data availability and Governance and Data Handling sections together emphasize that:

- only minimal metadata are imported into the Biobank LIMS,
- clinical and research data remain under existing governance structures,
- and centralized monitoring, redundancy, and 24/7 emergency response are designed to reduce, rather than increase, continuity risk.

I believe this now provides the general “risk and contingency” template requested, while keeping institution-specific details appropriately high-level.

**8. “Central question: why centralized auditing instead of simple replacement?”**

**Reviewer comment (paraphrased)**

 A critical reader might argue that simply replacing old units with improved technology would achieve the goals. The article should more clearly state what central auditing and governance add, how trade-offs are managed, and under what criteria the Biobank deviates from cost/energy savings to prioritize quality and continuity.

**Response**

 I agree that this is a central conceptual point, and I have strengthened it in several places:

- In the Introduction, as noted earlier, I now explicitly contrast governance-coupled changes with simple replacement: “Unlike simple replacement of outdated equipment, this model couples infrastructure upgrades with institutional governance, yielding sustainability, quality, and contingency gains not achievable through technology refresh alone.”

- In Governance and Data Handling and Results & discussion, I clarify the prioritization logic the reviewer requests:
  - Centralization is prioritized for long-term, hazardous, and irreplaceable materials where specimen quality and continuity are paramount.
  - High-frequency, short-turnaround materials remain decentralized even if this is less efficient energetically, because workflow and clinical requirements are prioritized.
  - Replacement decisions are made within a governance framework that balances sustainability with scientific and clinical needs.
- In the Conclusion, I explicitly re-cast the sustainability effort as simultaneously a quality initiative: “This case provides a practical framework for other academic biobanks seeking to balance financial stability with uncompromising quality and continuity, demonstrating that efficiency and rigor can be achieved together. Ultimately, this model demonstrates that sustainability initiatives can also serve as quality initiatives, improving research rigor and resiliency while reducing institutional risk.”

I hope these revisions make it clear that the project is not simply about newer equipment, but about a governance and cultural model that aligns infrastructure decisions with institutional stewardship of both environmental and scientific quality.

**9. “Data access and burden of case-by-case negotiation”**

**Reviewer comment (paraphrased)**

 The reviewer notes concern that requiring case-by-case data access negotiations may be a barrier for others seeking to replicate the approach.

**Response**

 I appreciate this point and have revised the Data availability section to clarify the rationale and to explain that the quantitative estimates in the manuscript are based on aggregate operational assessments intended to be reusable by others: “The Johns Hopkins institutional compliance offices advised that operational and infrastructure datasets should remain restricted due to security and confidentiality considerations. Quantitative estimates provided in this report are based on aggregate operational assessments intended to convey institutional impact without exposing sensitive infrastructure details.”

 I fully agree that replication should be as straightforward as possible. For that reason, the manuscript now emphasizes the framework (audit → governance → storage strategy → monitoring & contingency) and provides transferable categories (freezer age bands, percent of assets in aging categories, rough energy cost differential, and freezer-equivalent centralized capacity) that other institutions can estimate from their own data without needing access to Johns Hopkins–specific records.

 Once again, I thank the reviewer for the detailed and thoughtful critique. The revisions described above substantially expand the manuscript’s quantitative grounding, clarify the relative impact across sustainability, quality, and continuity, and more clearly articulate why a governance-driven, centralized approach offers benefits beyond simple equipment replacement. I hope the revised version now meets the reviewer’s expectations for a useful and generalizable case study template.

# Reviewer report: Reviewer response for version 1

**DOI:** 10.5256/f1000research.188763.r435167

**Published:** 2025-12-24

**Authors:** Amanda Rush

**Affiliations:** 1 The University of Sydney, Sydney, New South Wales, Australia

This manuscript describes a case study of freezer consolidation, with a view to improving both environmental and financial sustainability within a large institution.

 Major comments:

- It would be useful for the reader to have more detail on any changes in governance that were associated with shifting samples to the service centre. Did the migration of samples to the service centre have any other consequences for individual investigators to consider e.g. a minimum dataset, mandates for samples to be open access – or did all aspects of sample management and oversight remain the same for individuals, except for the storage location?
- It would also be interesting to learn more about any associated data migration, both in terms of LIMS, as well as other data associated with the samples. Did the latter (e.g. clinical data) remain with the investigators?
- The scale of the shift is unclear at this stage. While the manuscript states that 938 freezers were past their median life expectancy, it is unknown approximately what proportion of these failed from 2021 onwards. This has impacts on costs for the service centre. Was additional LN2 storage required to be purchased? Was the cost of the Varios incorporated into storage fees for investigators?
- The audit and consultation with stakeholders could have more detail added, in order for others to be able to adopt the approach. For example, it would be useful to know the proportion that migrated their collections vs met with the team to seek guidance on new freezers to purchase. What were the stakeholder responses when faced with shifting from -80C to LN2 storage? What information was sought from them?
- There is a time component to this work that is not acknowledged for others wishing to adopt the general approach – as freezers don’t fail regularly. Over approximately what time period would it be expected to reach a state where all freezers that were going to be migrated, have been migrated?
- It would be useful to have some evidence on approximate cost savings and energy savings that were brought about from the changes, in order to back up the claims made in the manuscript.
- At various stages in the manuscript, the reader is led to believe that sustainability refers to environmental sustainability and financial sustainability. It would be useful to explicitly detail if both of these meanings are intended by the title.

Minor comments:

- It would be useful to include a reference (if applicable) on the median life expectancy of the ULT freezers. Is this based on commercial advice or institutional data?
- There is some repetition in the narrative that could be removed and potentially replaced with a more in-depth description of the process, in order for the readers to assess whether it would be applicable for their own settings.

Is the case presented with sufficient detail to be useful for teaching or other practitioners?

Partly

Is the work clearly and accurately presented and does it cite the current literature?

Yes

If applicable, is the statistical analysis and its interpretation appropriate?

Not applicable

Are all the source data underlying the results available to ensure full reproducibility?

No

Are the conclusions drawn adequately supported by the results?

Partly

Is the background of the case’s history and progression described in sufficient detail?

Partly

Reviewer Expertise:

Human tissue biobanking

I confirm that I have read this submission and believe that I have an appropriate level of expertise to confirm that it is of an acceptable scientific standard, however I have significant reservations, as outlined above.

# Response

**Published:** 2025-12-30

**Authors:** Melissa Olson

**Affiliations:** Genetic Medicine, Johns Hopkins University School of Medicine, Baltimore, Maryland, USA

**Response to Reviewer (Dr. Amanda Rush)**

 I thank the reviewer for the thoughtful and constructive comments, and for recommending the manuscript as acceptable with reservations. Below I respond to each point and summarize the corresponding revisions.

**Major comments**

**1. Governance changes and consequences for investigators; open access vs “storage-only” changes**

**Reviewer comment (paraphrased)**

 More detail is needed on governance changes associated with shifting samples to the service center. Did migration to the service center require a minimum dataset, open-access mandates, or other changes to investigator control, or did management/oversight remain the same apart from storage location?

**Response**

 I agree that the governance implications should be explicit. I have clarified that centralization is a storage and stewardship intervention, not a change in scientific or data governance:

- In the Governance and Data Handling section, I now state: “Governance changes were designed to improve infrastructure while preserving investigator autonomy. Sample ownership and scientific decision-making remained fully with originating research teams, regardless of storage location. The Biobank assumed responsibility for storage quality, monitoring, chain-of-custody, and 24/7 emergency response, while associated clinical and research data remained with investigators or existing institutional systems. Only minimal metadata needed for traceability (e.g., specimen type, owner, hazard status, location, global identifier) were integrated into the Biobank LIMS.”

- In the Results & discussion, I further emphasize: “Centralization does, however, standardize stewardship expectations, including inventory documentation, access controls, and continuity planning, ensuring that biospecimens remain protected and discoverable even as personnel or research priorities change. This model strengthens stewardship of physical materials while preserving existing data-access controls and consent protections, ensuring that participant trust and regulatory compliance remain intact.”

No minimum dataset for scientific variables, no open-access requirement, and no change to consent or data-access rules were imposed as a consequence of migration; investigators retain full control over access, use, and sharing of their collections. The only governance shift is in infrastructure and monitoring, not in data rights.

**2. Data migration: LIMS vs clinical data**

**Reviewer comment (paraphrased)**

 More detail is requested on associated data migration – what moved into the Biobank LIMS and what remained with investigators (e.g., clinical data).

**Response**

 I appreciate this point and have made the data boundaries more explicit:

- As quoted above from “Governance and Data Handling”, I now specify: “Inventory records for all centralized collections are incorporated into the Biobank’s LIMS, supporting chain-of-custody, audit trails, and recovery planning. Associated clinical or research data remain with the investigator, consistent with consent governance and data stewardship policies. This separation ensures that enhancing physical storage does not alter data oversight or regulatory pathways.”- and- “Only minimal metadata needed for traceability (e.g., specimen type, owner, hazard status, location, global identifier) were integrated into the Biobank LIMS.”

Thus, data migration is deliberately limited to logistical metadata necessary for physical stewardship and continuity; clinical and research datasets remain under the originating teams’ systems and governance.

**3. Scale of the shift; failure of older freezers; LN₂ capacity and Vario costs**

**Reviewer comment (paraphrased)**

 The scale of the shift is unclear. While 938 freezers were past median life expectancy, it is not stated what proportion failed from 2021 onwards. Was additional LN₂ capacity purchased? Were Vario costs incorporated into storage fees?

**Response**

 I agree that readers need a clearer sense of scale and financial logic, while recognizing institutional constraints on disclosing detailed infrastructure inventories.

- In Results & discussion, I now quantify the stabilized centralized capacity: “Since 2021, centralized storage within the Biobank has stabilized the equivalent capacity of approximately 42 ultra-low temperature freezers, eliminating the need for laboratories to maintain or replace those units and reducing institutional exposure to aging, high-risk infrastructure.”

This communicates the scale of the realized shift, without listing individual units or locations.

- Regarding freezer failures among the 938 units beyond median life expectancy, the initiative was implemented prospectively and aligned with life-cycle events rather than as a retrospective failure-rate study. For that reason, I have not added a specific proportion of failures, as these data were not systematically captured in a way that would support robust reporting at the level of detail requested. Instead, I emphasize the conceptual linkage: “Adoption of centralized storage is inherently a gradual process, tied to equipment failure cycles, research timelines, voluntary participation and cultural adaptation. Most ULT freezers in academic settings are only replaced at end-of-life, and failure events are unpredictable. Accordingly, achieving full transition across eligible collections is expected to occur over multiple years rather than rapid turnover, with each unit failure representing an opportunity to migrate to sustainable, governed storage.”

- With respect to infrastructure costs, additional LN₂ and Vario capacity were indeed required and were added in a staged fashion as migration opportunities arose. Rather than detailing internal capital allocations, I describe this in terms of service-center economics:

“Specimen deposits were managed as service requests, with annual storage charges applied to ensure cost neutrality and long-term sustainability of the service center.”

 The cost of LN₂ and Vario operation is built into the standard storage fee structure as part of routine rate-setting and cost-recovery, rather than being passed to individual investigators as a separate technology surcharge. I did not include more granular cost breakdowns but I have strengthened the explanation of the cost-recovery logic.

**4. Audit and consultation details; stakeholder responses to LN₂ vs –80 °C; migration vs guidance**

**Reviewer comment (paraphrased)**

 More detail on the audit and stakeholder engagement would help others adopt the approach. For example: proportion of stakeholders who migrated collections vs those who just sought guidance on new freezers; what were responses to shifting from –80 °C to LN₂; what information was sought from investigators?

**Response**

 I appreciate the emphasis on practical, transferable details. While I cannot provide precise proportions for each stakeholder pathway (these were not formally quantified as a research outcome), I have expanded the narrative on engagement and typical responses.

- In Methods/Approach, I clarify the engagement structure: “Because this initiative was intentionally aligned with natural equipment life cycles, investigators are required to evaluate community-based biobanking within the Johns Hopkins Biobank before purchasing replacements when older laboratory freezers failed. This ‘shared resources before new purchases’ policy anchors the governance element of our model (Figure 1). Outreach campaigns, faculty meetings, and symposia reinforced awareness of centralized options and the advantages of shared stewardship. When centralized LN₂ or Vario storage was not feasible, Biobank staff engaged in direct consultation with investigators to ensure that any new freezer purchases aligned with institutional efficiency and sustainability goals.”

- In the Results & discussion, I elaborate on the nature of these consultations and the heterogeneity of responses. I emphasize that:
  - Some groups elected to migrate cohorts into central LN₂ or Vario storage.
  - Others used the consultation primarily to guide the purchase of more efficient departmental ULT freezers when decentralization was operationally necessary.
  - Common concerns included perceived access delays, familiarity and comfort with LN₂, and the suitability of colder storage for particular assay pipelines.

Given the absence of systematically collected proportions, I stop short of assigning percentages, but the pathway structure and typical concerns are now clearly described so that other institutions can consider analogous workflows.

**5. Time component: how long until the “steady state” of migration?**

**Reviewer comment (paraphrased)**

 There is an important time component, since freezers do not fail regularly. Over approximately what time period would it be expected to reach a state where all freezers that were going to be migrated have been migrated?

**Response**

 I fully agree that the time horizon matters. The manuscript now explicitly frames the initiative as multi-year and tied to life-cycle replacement:

- As noted above and now emphasized in Results & discussion: “Adoption of centralized storage is inherently a gradual process, tied to equipment failure cycles, research timelines, voluntary participation and cultural adaptation. Most ULT freezers in academic settings are only replaced at end-of-life, and failure events are unpredictable. Accordingly, achieving full transition across eligible collections is expected to occur over multiple years rather than rapid turnover, with each unit failure representing an opportunity to migrate to sustainable, governed storage.”

Given a median life expectancy of ~8.5 years and the age distribution observed in the audit, a substantial fraction of units eligible for replacement are expected to cycle through within a 5–10 year horizon after policy implementation, but exact forecasting is not feasible because of heterogeneous usage patterns and institutional decisions. I therefore frame the time aspect as a principled multi-year progression rather than a precise deadline.

**6. Evidence for cost and energy savings**

**Reviewer comment (paraphrased)**

 It would be useful to have approximate cost and energy savings to support the claims.

**Response**

 I agree, and I have strengthened this component in the Results & discussion: “Within the School of Medicine, these aging freezers consume an estimated additional $800K annually in avoidable electricity and HVAC load, based on industry-validated energy modeling (including manufacturer performance curves and published ULT power consumption data). This infrastructure inefficiency is disproportionately driven by decentralized research storage, which accounts for ~80% of ULT assets across campus.”

 I pair this with the quantified centralized capacity: “Since 2021, centralized storage within the Biobank has stabilized the equivalent capacity of approximately 42 ultra-low temperature freezers, eliminating the need for laboratories to maintain or replace those units and reducing institutional exposure to aging, high-risk infrastructure.”

 Together, these figures provide a ballpark sense of avoided cost and energy demand, without disclosing smaller-scale operational details that are considered sensitive.

**7. Clarifying what “sustainability” means (environmental and financial)**

**Reviewer comment (paraphrased)**

 Sustainability appears to refer to both environmental and financial aspects. It would be useful to explicitly state whether both meanings are intended.

**Response**

 Thank you for highlighting this. I have clarified in the Introduction that both aspects are intended: “This creates a dual challenge. On one hand, institutions are under increasing pressure to reduce the environmental footprint of research infrastructure in alignment with broader sustainability commitments. On the other, biobanks must maintain uncompromising standards of quality and continuity to protect biospecimens, meet regulatory requirements, and preserve trust with research participants. Efforts to reduce energy use or rationalize equipment therefore cannot come at the expense of specimen protection or emergency readiness, nor can cost-recovery models undermine long-term stewardship.”

 In the Conclusion, I make the dual nature explicit: “This case provides a practical framework for other academic biobanks seeking to balance financial stability with uncompromising quality and continuity, demonstrating that efficiency and rigor can be achieved together. Ultimately, this model demonstrates that sustainability initiatives can also serve as quality initiatives, improving research rigor and resiliency while reducing institutional risk.”

 Thus, throughout the manuscript **“sustainability” explicitly encompasses both environmental (energy, cooling load, carbon footprint) and financial (cost-recovery, infrastructure efficiency) dimensions.

**Minor comments**

**1. Reference or basis for median life expectancy of 8.5 years**

**Reviewer comment (paraphrased)**

 Please clarify the basis for the 8.5-year median life expectancy for ULT freezers.

**Response**

 I have clarified that this figure is grounded in published work and institutional experience. In the Methods section, I now cite the relevant literature.

**2. Repetition in the narrative vs depth of process description**

**Reviewer comment (paraphrased)**

 There is some repetition in the narrative that could be removed and potentially replaced with more in-depth process description.

**Response**

 I appreciate this stylistic suggestion and have streamlined several repeated elements, particularly:

- Reducing repeated general statements about the value of biobanking and the basic description of LN₂/Vario advantages.
- Consolidating overlapping phrases about “balancing sustainability, quality, and continuity” so they appear once in the Introduction and again in a more analytical way in the Results & discussion.

Space gained from these edits has been used to:

- Expand the “Governance and Data Handling” section with clearer detail on investigator autonomy, metadata scope, and data separation.
- Strengthen the Results & discussion on the timing of benefits, the cultural shift from freezer ownership to specimen stewardship, and practical risks and mitigation during transition.

I hope this improves readability while giving readers a richer description of the process they may wish to adapt.

 Once again, I thank the reviewer for the thoughtful and constructive feedback. The revisions in response to these comments have, I believe, improved the clarity, transferability, and evidentiary support of the case study, particularly around governance, time horizon, and the dual environmental and financial dimensions of sustainability.
